# Benzyl 3-[(*E*)-furfuryl­idene]dithio­carbazate

**DOI:** 10.1107/S160053680801307X

**Published:** 2008-05-10

**Authors:** Shang Shan, Yu-Liang Tian, Shan-Heng Wang, Wen-Long Wang, Ying-Li Xu

**Affiliations:** a College of Chemical Engineering and Materials Science, Zhejiang University of Technology, People’s Republic of China

## Abstract

In the title compound, C_13_H_12_N_2_OS_2_, the mol­ecule assumes an *E* configuration, with the furan ring and dithio­carbazate units located on opposite sides of the N=C double bond. In the crystal structure, mol­ecules are linked *via* two inter­molecular N—H⋯S hydrogen bonds to form centrosymmetric dimers.

## Related literature

For general background, see: Okabe *et al.* (1993 ▶). For related structures, see: Shan *et al.* (2006 ▶, 2008 ▶). For the synthesis and background, see: Hu *et al.* (2001 ▶).
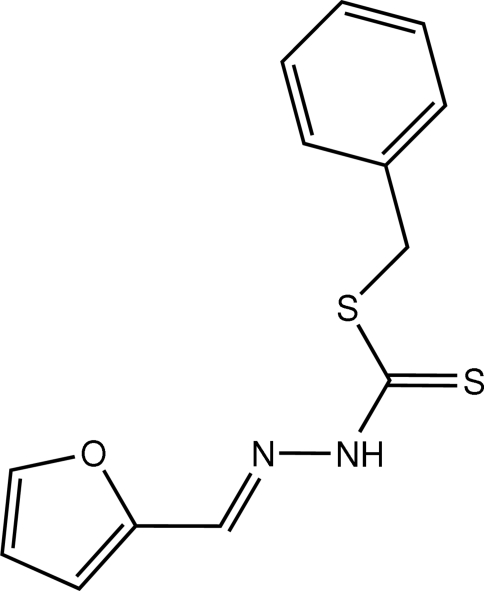


## Experimental

### 

#### Crystal data

C_13_H_12_N_2_OS_2_
*M* *_r_* = 276.37
Triclinic, 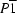

*a* = 4.8331 (11) Å
*b* = 12.040 (3) Å
*c* = 12.549 (3) Å
α = 108.203 (7)°
β = 99.704 (9)°
γ = 97.910 (8)°
*V* = 669.5 (3) Å^3^
*Z* = 2
Mo *K*α radiation
μ = 0.39 mm^−1^
*T* = 295 (2) K
0.42 × 0.36 × 0.32 mm

#### Data collection

Rigaku R-AXIS RAPID IP diffractometer
Absorption correction: none
7084 measured reflections
2324 independent reflections
1799 reflections with *I* > 2σ(*I*)
*R*_int_ = 0.024

#### Refinement

*R*[*F* ^2^ > 2σ(*F* ^2^)] = 0.033
*wR*(*F* ^2^) = 0.090
*S* = 1.07
2324 reflections
163 parameters
H-atom parameters constrained
Δρ_max_ = 0.14 e Å^−3^
Δρ_min_ = −0.17 e Å^−3^

### 

Data collection: *PROCESS-AUTO* (Rigaku, 1998 ▶); cell refinement: *PROCESS-AUTO*; data reduction: *CrystalStructure* (Rigaku/MSC, 2002 ▶); program(s) used to solve structure: *SIR92* (Altomare *et al.*, 1993 ▶); program(s) used to refine structure: *SHELXL97* (Sheldrick, 2008 ▶); molecular graphics: *ORTEP-3 for Windows* (Farrugia, 1997 ▶); software used to prepare material for publication: *WinGX* (Farrugia, 1999 ▶).

## Supplementary Material

Crystal structure: contains datablocks I, global. DOI: 10.1107/S160053680801307X/hb2727sup1.cif

Structure factors: contains datablocks I. DOI: 10.1107/S160053680801307X/hb2727Isup2.hkl

Additional supplementary materials:  crystallographic information; 3D view; checkCIF report

## Figures and Tables

**Table 1:** Hydrogen-bond geometry (Å, °)

*D*—H⋯*A*	*D*—H	H⋯*A*	*D*⋯*A*	*D*—H⋯*A*
N2—H2*N*⋯S1^i^	0.86	2.56	3.3761 (19)	158

Symmetry code: (i) 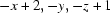.

**Fig. 1.**
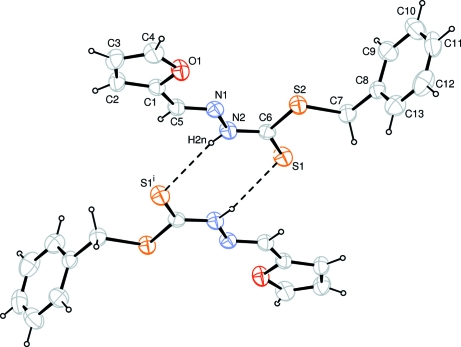
The inversion dimer in the crystal of (I) drawn with 30% probability displacement ellipsoids (arbitrary spheres for H atoms). Dashed lines indicate hydrogen bonding [symmetry code: (i) 2 - x,-y,1 - z].

## supplementary crystallographic information

### Comment

Hydrazone and its derivatives have shown the potential application in biological field (Okabe *et al.*, 1993). As part of our ongoing investigation on anti-cancer compounds (Hu *et al.*, 2001), the title compound, (I), has been prepared in our laboratory and its crystal structure is presented here.

The N1—C5 distance indicates a typical C=N double bond. The furan and dithiocarbazate moieties are located on the opposite positions of the C=N bond, thus the molecule assumes an E-configuration, which agrees with that found in methyl (β-*N*-phenylmethylene)dithiocarbazate (Shan *et al.*, 2006).

In the molecule of (I), the furan ring is slightly twisted with respect to the dithiocarbazate plane with a dihedral angle of 7.58 (14)°, whereas the phenyl ring of the thioester group is nearly perpendicular to the dithiocarbazate plane with a dihedral angle of 85.51 (5)°. This is similar to that found in a related structure, benzyl 3-[(*E*)-phenylmethylene]dithiocarbazate (Shan *et al.*, 2008).

In the crystal of (I), adjacent molecules are linked by intermolecular N—H···S hydrogen bonding into inversion dimers (Fig. 1 and Table 1).

### Experimental

Benzyl dithiocarbazate was synthesized as described previously (Hu *et al.*, 2001). Benzyl dithiocarbazate (1.98 g, 10 mmol) and furfural (0.96 g, 10 mmol) were dissolved in ethanol (40 ml) and the solution was refluxed for 12 h. A yellow crystalline product appeared after cooling to room temperature; it was separated and washed with cold water three times. Yellow prisms of (I) were obtained by recrystallization from an ethanol solution.

### Refinement

The H atoms were placed in calculated positions with C—H = 0.97 (methylene), 0.93Å (aromatic) and N—H = 0.86 Å, and refined as riding with *U*_iso_(H) = 1.2*U*_eq_(C,*N*)

### Crystal data

**Table:**

C_13_H_12_N_2_OS_2_	*Z* = 2
*M**_r_* = 276.37	*F*_000_ = 288
Triclinic, *P*1	*D*_x_ = 1.371 Mg m^−^^3^
Hall symbol: -P 1	Mo *K*α radiation λ = 0.71073 Å
*a* = 4.8331 (11) Å	Cell parameters from 3836 reflections
*b* = 12.040 (3) Å	θ = 1.8–25.0º
*c* = 12.549 (3) Å	µ = 0.39 mm^−^^1^
α = 108.203 (7)º	*T* = 295 (2) K
β = 99.704 (9)º	Prism, yellow
γ = 97.910 (8)º	0.42 × 0.36 × 0.32 mm
*V* = 669.5 (3) Å^3^	

### Data collection

**Table:**

Rigaku R-AXIS RAPID IP diffractometer	2324 independent reflections
Radiation source: fine-focus sealed tube	1799 reflections with *I* > 2σ(*I*)
Monochromator: graphite	*R*_int_ = 0.024
Detector resolution: 10.00 pixels mm^-1^	θ_max_ = 25.0º
*T* = 295(2) K	θ_min_ = 1.8º
ω scans	*h* = −5→5
Absorption correction: none	*k* = −14→13
7084 measured reflections	*l* = −14→14

### Refinement

**Table:**

Refinement on *F*^2^	Secondary atom site location: difference Fourier map
Least-squares matrix: full	Hydrogen site location: inferred from neighbouring sites
*R*[*F*^2^ > 2σ(*F*^2^)] = 0.033	H-atom parameters constrained
*wR*(*F*^2^) = 0.090	*w* = 1/[σ^2^(*F*_o_^2^) + (0.0446*P*)^2^ + 0.0525*P*] where *P* = (*F*_o_^2^ + 2*F*_c_^2^)/3
*S* = 1.07	(Δ/σ)_max_ < 0.001
2324 reflections	Δρ_max_ = 0.14 e Å^−^^3^
163 parameters	Δρ_min_ = −0.17 e Å^−^^3^
Primary atom site location: structure-invariant direct methods	Extinction correction: none

### Special details

**Table:**

Geometry. All e.s.d.'s (except the e.s.d. in the dihedral angle between two l.s. planes) are estimated using the full covariance matrix. The cell e.s.d.'s are taken into account individually in the estimation of e.s.d.'s in distances, angles and torsion angles; correlations between e.s.d.'s in cell parameters are only used when they are defined by crystal symmetry. An approximate (isotropic) treatment of cell e.s.d.'s is used for estimating e.s.d.'s involving l.s. planes.
Refinement. Refinement of *F*^2^ against ALL reflections. The weighted *R*-factor *wR* and goodness of fit *S* are based on *F*^2^, conventional *R*-factors *R* are based on *F*, with *F* set to zero for negative *F*^2^. The threshold expression of *F*^2^ > σ(*F*^2^) is used only for calculating *R*-factors(gt) *etc*. and is not relevant to the choice of reflections for refinement. *R*-factors based on *F*^2^ are statistically about twice as large as those based on *F*, and *R*- factors based on ALL data will be even larger.

### Fractional atomic coordinates and isotropic or equivalent isotropic displacement parameters (Å^2^)

**Table:**

	*x*	*y*	*z*	*U*_iso_*/*U*_eq_	
S1	1.03544 (13)	−0.02756 (5)	0.32307 (4)	0.0669 (2)	
S2	0.84716 (12)	0.18647 (4)	0.27822 (4)	0.06143 (19)	
O1	0.4143 (3)	0.41877 (12)	0.56065 (11)	0.0662 (4)	
N1	0.6602 (3)	0.22213 (13)	0.48240 (13)	0.0533 (4)	
N2	0.7837 (3)	0.12309 (13)	0.45371 (13)	0.0569 (4)	
H2N	0.7943	0.0807	0.4976	0.068*	
C1	0.4147 (4)	0.33502 (16)	0.61307 (15)	0.0527 (5)	
C2	0.2854 (5)	0.36601 (19)	0.70185 (17)	0.0640 (5)	
H2	0.2592	0.3242	0.7516	0.077*	
C3	0.1967 (5)	0.47383 (19)	0.70534 (19)	0.0711 (6)	
H3	0.1011	0.5168	0.7574	0.085*	
C4	0.2776 (5)	0.50159 (19)	0.61914 (19)	0.0720 (6)	
H4	0.2452	0.5687	0.6011	0.086*	
C5	0.5457 (4)	0.23599 (17)	0.56929 (16)	0.0548 (5)	
H5	0.5479	0.1793	0.6054	0.066*	
C6	0.8870 (4)	0.09223 (16)	0.35868 (15)	0.0510 (5)	
C7	1.0091 (5)	0.11971 (19)	0.15901 (17)	0.0647 (5)	
H7A	1.2107	0.1229	0.1872	0.078*	
H7B	0.9145	0.0368	0.1189	0.078*	
C8	0.9740 (4)	0.19026 (17)	0.07913 (16)	0.0560 (5)	
C9	0.7422 (5)	0.1560 (2)	−0.01297 (19)	0.0764 (6)	
H9	0.6064	0.0877	−0.0258	0.092*	
C10	0.7056 (7)	0.2192 (3)	−0.0861 (2)	0.0916 (8)	
H10	0.5469	0.1939	−0.1476	0.110*	
C11	0.9002 (8)	0.3183 (3)	−0.0689 (3)	0.0931 (9)	
H11	0.8750	0.3616	−0.1182	0.112*	
C12	1.1348 (7)	0.3554 (2)	0.0210 (3)	0.0979 (9)	
H12	1.2695	0.4235	0.0325	0.117*	
C13	1.1712 (5)	0.2906 (2)	0.0954 (2)	0.0795 (6)	
H13	1.3306	0.3159	0.1565	0.095*	

### Atomic displacement parameters (Å^2^)

**Table:**

	*U*^11^	*U*^22^	*U*^33^	*U*^12^	*U*^13^	*U*^23^
S1	0.0906 (4)	0.0557 (3)	0.0675 (3)	0.0399 (3)	0.0222 (3)	0.0265 (2)
S2	0.0790 (4)	0.0596 (3)	0.0632 (3)	0.0369 (3)	0.0240 (3)	0.0313 (2)
O1	0.0945 (11)	0.0586 (8)	0.0640 (8)	0.0373 (7)	0.0296 (7)	0.0313 (7)
N1	0.0610 (10)	0.0490 (9)	0.0560 (9)	0.0254 (8)	0.0127 (8)	0.0206 (7)
N2	0.0722 (11)	0.0519 (9)	0.0595 (9)	0.0302 (8)	0.0188 (8)	0.0275 (7)
C1	0.0573 (12)	0.0517 (11)	0.0549 (10)	0.0174 (9)	0.0118 (9)	0.0244 (9)
C2	0.0741 (14)	0.0667 (13)	0.0656 (12)	0.0244 (11)	0.0285 (11)	0.0316 (10)
C3	0.0787 (15)	0.0672 (14)	0.0736 (14)	0.0314 (12)	0.0303 (12)	0.0187 (11)
C4	0.0920 (17)	0.0575 (13)	0.0773 (14)	0.0389 (12)	0.0243 (12)	0.0253 (11)
C5	0.0623 (12)	0.0526 (11)	0.0585 (11)	0.0213 (9)	0.0138 (9)	0.0275 (9)
C6	0.0525 (11)	0.0483 (11)	0.0547 (10)	0.0173 (9)	0.0079 (8)	0.0201 (8)
C7	0.0737 (14)	0.0654 (13)	0.0702 (12)	0.0341 (11)	0.0265 (11)	0.0303 (10)
C8	0.0629 (13)	0.0559 (12)	0.0600 (11)	0.0263 (10)	0.0251 (10)	0.0229 (9)
C9	0.0843 (17)	0.0717 (15)	0.0764 (14)	0.0172 (12)	0.0151 (13)	0.0309 (12)
C10	0.117 (2)	0.100 (2)	0.0697 (15)	0.0416 (18)	0.0182 (14)	0.0385 (15)
C11	0.132 (3)	0.103 (2)	0.0922 (19)	0.065 (2)	0.0647 (19)	0.0602 (17)
C12	0.103 (2)	0.0752 (17)	0.142 (3)	0.0230 (16)	0.062 (2)	0.0551 (18)
C13	0.0761 (16)	0.0753 (16)	0.0940 (16)	0.0195 (13)	0.0238 (13)	0.0346 (13)

### Geometric parameters (Å, °)

**Table:**

S1—C6	1.6686 (18)	C5—H5	0.9300
S2—C6	1.7477 (19)	C7—C8	1.507 (3)
S2—C7	1.820 (2)	C7—H7A	0.9700
O1—C4	1.363 (2)	C7—H7B	0.9700
O1—C1	1.365 (2)	C8—C13	1.369 (3)
N1—C5	1.280 (2)	C8—C9	1.378 (3)
N1—N2	1.381 (2)	C9—C10	1.369 (3)
N2—C6	1.336 (2)	C9—H9	0.9300
N2—H2N	0.8600	C10—C11	1.348 (4)
C1—C2	1.345 (3)	C10—H10	0.9300
C1—C5	1.428 (3)	C11—C12	1.368 (4)
C2—C3	1.412 (3)	C11—H11	0.9300
C2—H2	0.9300	C12—C13	1.395 (4)
C3—C4	1.329 (3)	C12—H12	0.9300
C3—H3	0.9300	C13—H13	0.9300
C4—H4	0.9300		
			
C6—S2—C7	102.07 (9)	C8—C7—S2	107.15 (13)
C4—O1—C1	106.12 (15)	C8—C7—H7A	110.3
C5—N1—N2	114.92 (16)	S2—C7—H7A	110.3
C6—N2—N1	120.90 (15)	C8—C7—H7B	110.3
C6—N2—H2N	119.6	S2—C7—H7B	110.3
N1—N2—H2N	119.6	H7A—C7—H7B	108.5
C2—C1—O1	109.46 (17)	C13—C8—C9	117.6 (2)
C2—C1—C5	131.99 (18)	C13—C8—C7	121.2 (2)
O1—C1—C5	118.55 (16)	C9—C8—C7	121.1 (2)
C1—C2—C3	107.23 (19)	C10—C9—C8	122.0 (2)
C1—C2—H2	126.4	C10—C9—H9	119.0
C3—C2—H2	126.4	C8—C9—H9	119.0
C4—C3—C2	106.14 (18)	C11—C10—C9	119.8 (3)
C4—C3—H3	126.9	C11—C10—H10	120.1
C2—C3—H3	126.9	C9—C10—H10	120.1
C3—C4—O1	111.05 (19)	C10—C11—C12	120.3 (3)
C3—C4—H4	124.5	C10—C11—H11	119.9
O1—C4—H4	124.5	C12—C11—H11	119.9
N1—C5—C1	122.66 (18)	C11—C12—C13	119.7 (3)
N1—C5—H5	118.7	C11—C12—H12	120.1
C1—C5—H5	118.7	C13—C12—H12	120.1
N2—C6—S1	121.22 (14)	C8—C13—C12	120.5 (2)
N2—C6—S2	114.01 (13)	C8—C13—H13	119.7
S1—C6—S2	124.76 (11)	C12—C13—H13	119.7

### Hydrogen-bond geometry (Å, °)

**Table:**

*D*—H···*A*	*D*—H	H···*A*	*D*···*A*	*D*—H···*A*
N2—H2N···S1^i^	0.86	2.56	3.3761 (19)	158

Symmetry codes: (i) −*x*+2, −*y*, −*z*+1.
